# Data Citation in Neuroimaging: Proposed Best Practices for Data Identification and Attribution

**DOI:** 10.3389/fninf.2016.00034

**Published:** 2016-08-12

**Authors:** Leah B. Honor, Christian Haselgrove, Jean A. Frazier, David N. Kennedy

**Affiliations:** ^1^Lamar Soutter Library, University of Massachusetts Medical SchoolWorcester MA, USA; ^2^Eunice Kennedy Shriver Center, University of Massachusetts Medical SchoolWorcester MA, USA; ^3^Child and Adolescent NeuroDevelopment Initiative, Department of Psychiatry, University of Massachusetts Medical SchoolWorcester MA, USA

**Keywords:** data citation, data attribution, credit, data repository, data sharing

## Abstract

Data sharing and reuse, while widely accepted as good ideas, have been slow to catch on in any concrete and consistent way. One major hurdle within the scientific community has been the lack of widely accepted standards for citing that data, making it difficult to track usage and measure impact. Within the neuroimaging community, there is a need for a way to not only clearly identify and cite datasets, but also to derive new aggregate sets from multiple sources while clearly maintaining lines of attribution. This work presents a functional prototype of a system to integrate Digital Object Identifiers (DOI) and a standardized metadata schema into a XNAT-based repository workflow, allowing for identification of data at both the project and image level. These item and source level identifiers allow any newly defined combination of images, from any number of projects, to be tagged with a new group-level DOI that automatically inherits the individual attributes and provenance information of its constituent parts. This system enables the tracking of data reuse down to the level of individual images. The implementation of this type of data identification system would impact researchers and data creators, data hosting facilities, and data publishers, but the benefit of having widely accepted standards for data identification and attribution would go far toward making data citation practical and advantageous.

## Introduction

Amid growing institutional, national, and international pressure, data sharing is becoming an accepted and increasingly mandated component of the research process. Whether this means creating and submitting data to open access repositories, publishing full datasets as Supplemental Materials to a paper, or sharing data on a researcher-to-researcher level, the goal is always the same: to increase the availability and discoverability of research in the hope of widening its reach and improving its impact. With burgeoning concerns about the reproducibility of science, the urgency for developing “best practices” for generating and including the data sources associated with a scientific publication has grown dramatically in the recent years (Ioannidis, 2005, 2014; Button et al., 2013)^1^.

A number of national and international efforts have begun to attempt to address the guiding principles, standards, and policies that are essential to expanding data sharing across the spectrum of researchers, publishers, and funders. A recent paper by Wilkinson et al. (2016) promotes the FAIR Data Principles (Findability, Accessibility, Interoperability, and Reusability). The Research Data Alliance (RDA) Data Citation working group has provided recommendations regarding citation of evolving data^2^ that supports a dynamic, query centric view of data sets. Additionally, a Joint Declaration of Data Citation Principles (JDDCP) has emerged from the FORCE11 community^3^ (Starr et al., 2015) that promotes the dual necessity of human understandable and machine actionable methods of data citation. Together, these numerous recommendations and principles still need to be implemented within the specific data hosting repositories, and utilized by the authors, and publishers, in a fashion that is tailored to the specific needs of each community.

An author derives “credit” for their publication through its listing on their curriculum vitae (course of life) which can, in turn, typically be corroborated by accepted publication indexing services. The “impact” of a publication is inferred from factors like the number of citations the publication receives, and the perceived impact of the source journal of the publication, etc., Citations are the method by which the authors of a paper acknowledge credit to another publication for supporting (or contrasting) ideas, concepts, or observations. These impact factors are similarly monitored by accepted indexing services. This publication citation and attribution system works because of accepted standards for the identification of a publication, and the protocol that is followed to enable one publication to attribute another. For publications, the Digital Object Identifier (DOI) provides the accepted identification standard and specifies the necessary metadata to support credit and attribution. The accepted protocol for attribution is for a publication to include in-text citations and a “References” section that uniquely lists these cited publications. From this infrastructure and accepted practices by the publishers, the indexing services can identify unique publications, and the citations between them.

However, in contrast to “credit” for a publication, there currently lacks an efficient and accepted means of deriving credit for shared data. This is due, in part, to a lack of standards for identifying and attributing the use of shared data. This is a major sociological hurdle that still needs to be overcome in order to get researchers to fully commit to the effort required for data sharing. While data is slowly becoming recognized as a first-class research object, deserving of formal citation on its own (Mooney, 2011), there is no widely accepted method to ensure proper attribution is given, nor any way to quantitatively measure the impact of shared data. In this report, we review the data citation and attribution problem and propose a set of “best practices” for the identification and citation of neuroimaging data in a context that will ensure proper attribution and credit is maintained when data is reused in subsequent studies. Within neuroscience overall, the neuroimaging community is particularly well suited to spearhead adoption of a progressive data credit and citation system due to the sheer volume of data that exists, coupled with a reasonably advanced set of standards for data description and robust community repositories. It is hoped that, with researchers, institutions, and data repositories following such practices, eventually data citations can carry the same impact and prestige as references to published research papers within the scientific community. The following presents a vision of this credit and identification system, and the possibilities for further development of this effort.

## Background

Within the neuroimaging community, creating and populating shared image repositories has become common (Eickhoff et al., 2016). There are multiple examples of image collections, such as the Alzheimers Disease Neuroimaging Initiative (Mueller et al., 2005)^4^, the National Database of Autism Research (Hall et al., 2012)^5^, 1000 Functional Connectomes (Biswal et al., 2010)^6^, Autism Brain Image Data Exchange (Di Martino et al., 2014)^7^, ADHD-200 (Fair et al., 2012)^8^, etc., However, the levels of access among these initiatives can run the gamut from completely open and accessible to available only with institutional permissions, and there are no community-wide minimally acceptable standards for submission regarding format or supporting documentation. This heterogeneity can lead to frustration in trying to obtain raw data, discouraging reuse and replication, which are the goals of open science.

The description of data used in support of a research publication also presents problems when trying to track use and measure impact. Often manipulations, exclusions, or other modifications to a dataset are only described in a methods section, making them subject to individual interpretation. The intent of opening access to not only published papers but also to the supporting data is to increase the reproducibility and transparency of the science, but this can only be done if the underlying data used can be unambiguously defined. Additionally, researcher acceptance of the ideals, as applicable to human data, of “open data”^9^, is essential for its effectiveness. Promoting reuse of data will only be successful when data creators are confident that they will receive not only recognition and appreciation for their work, but measurable credit. This cannot be only in the form of acknowledgements or inclusion in lists of contributors (Mooney and Newton, 2012), but in standardized citations that can be counted and quantified as meaningful contributions to the scientific field.

In trying to create a best practice for how data could be cited, shared, and credited correctly within the neuroimaging community, three main areas of concern become evident: (1) how to uniquely identify data, and to what level of granularity; (2) how to adequately cite datasets, and make those citations visible, quantifiable and reusable; and (3) how to ensure the chain of proper attribution and credit is maintained, even in the case when new datasets are created from multiple sources. These objectives are addressed in the context of this best practices proposal.

## Approach

In the following sections we define the design considerations and reference implementation of a data citation framework. This is followed by a discussion of the implications for such a system, if it were put into routine use. We are assuming a generalized data structure: a subject has numerous **observations**. A **study** is typically comprised of a selected subset of observations from a group of subjects, chosen to answer a particular question of interest.

These observations could be behavioral measures, clinical observations, or biophysical measurements, such as an MRI image or an electroencephalograph. In the context of neuroimaging, the most common observation is an image. Each image may itself be comprised of numerous slices (covering a volume of space), timepoints (for dynamic imaging, such as functional MRI), or other encoding variables (such as diffusion weighting, spin labeling, etc.). Images are used as the prototype observation for the reference implementation reported below, though it is intended that the principles be widely generalizable regardless of the type of observation.

Studies are shared or published in a repository, at the discretion of the researchers, either to provide the supporting data for a paper, or to fulfill a grant-related data sharing requirement, etc., The hosts of this published data could be a publically-funded repository (such as NITRC Kennedy et al., 2016,^10^), a commercial repository (such as Dryad Evans, 2016,^11^ or FigShare^12^), or an institutional data repository (such as eScholarship^@^UMMS^13^). High-level information about the shared data within any repository needs to be openly available, accessible, and queryable, in a fashion analogous to a publication's ability to be discovered and indexed by PubMed, despite numerous journals, publishers, and varied access permissions that exist. The incorporation of a proper citation and credit scheme, therefore, requires a coordinated effort amongst researchers, their institutions, funding agencies, the public and private data hosting efforts, and the publishing community.

### What to cite?

Our approach is predicated on the idea that the most basic data-sharing element which should be indexed is that which could be meaningfully shared independently. In the neuroimaging context, an image from a subject (i.e., a T1-weighted volumetric scan) has meaning, whereas a specific slice of that scan (or single timepoint within a dynamic scan) would not, without the context provided by the rest of the slices (or timepoints). For this reason, the authors designated the image as the basic building block of the citation and credit system. By assigning a unique identifier at this level, various meaningful collections of these images can be assembled.

### Collections

**Figure 1:**
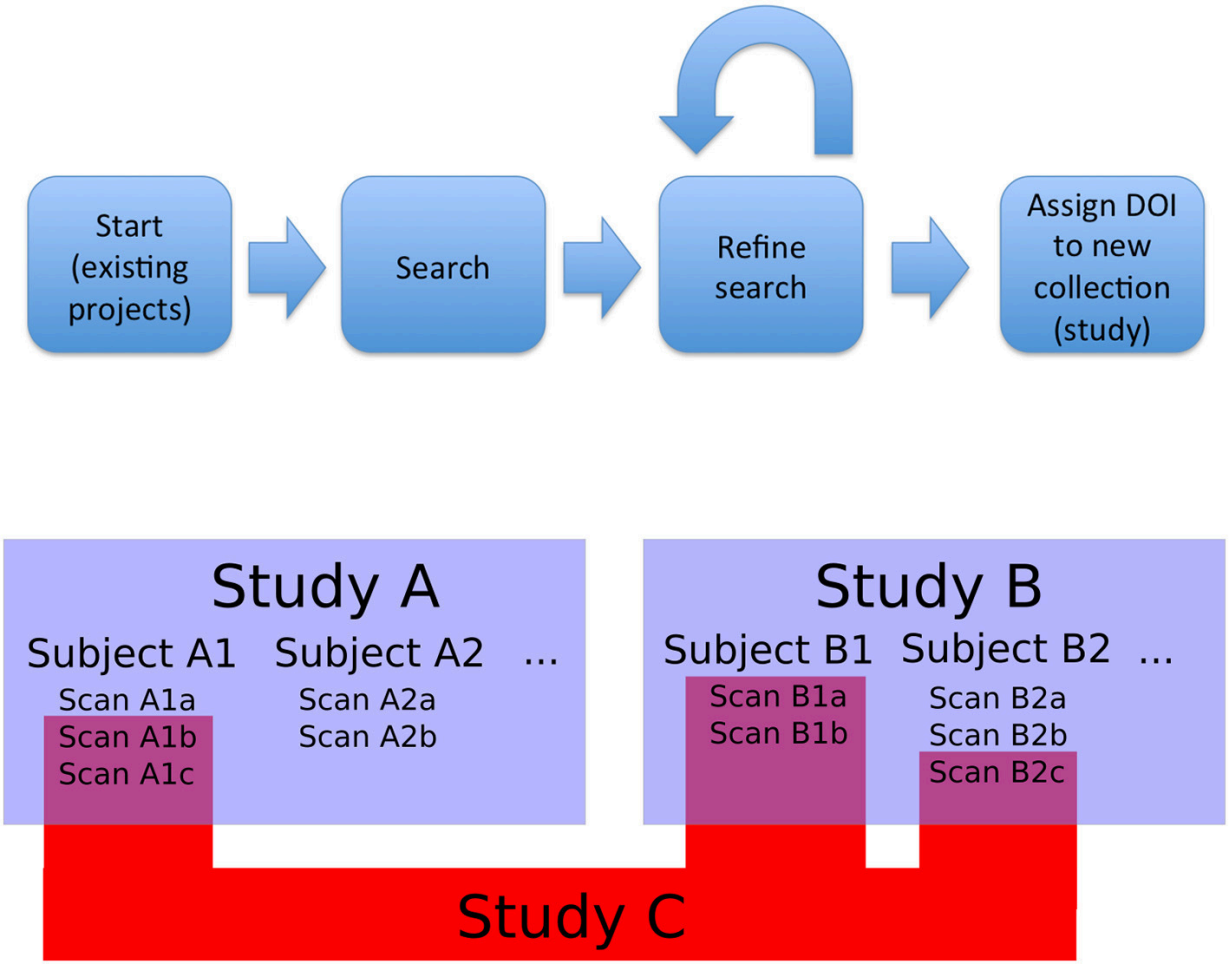
**Overview and workflow**. Bottom: studies, subjects, and observations (images). Studies A and B are projects that released data; they contain subjects and images. Study C is a functional study, made up of images from existing studies. In this case, Study C is made up of images from Study A and Study B (the component images are represented by RelatedIdentifier fields with relationType = HasPart in the DataCite schema, and the source projects are represented by RelatedIdentifier fields with relationType = IsDerivedFrom). As a new and useful collection of images, Study C can be assigned a DOI. Top: The overall flow for data selection and tagging. Data for a novel study is selected by searching existing studies (Study A and B). This search can be refined at a fine-grained level: individual images can be added or removed to the search results to create an arbitrary collection of images. The resulting collection is then tagged with a DOI and can be referenced from a publication that uses it. Existing projects (i.e., Study A and B) with constituent images can be queried and grouped into new collections (i.e., Study C) that retain attribution to the collection members.

The first type of collection we considered was the **project** (or study). In this case, the members of the collection are declared by the authors and the data tends to be stored together in a given repository. Second, we need to support ability to create new datasets, that could combine images from multiple projects (based upon search, the act of querying a particular data host for objects that meet a specific set of criteria, or other aggregation). This presents a significant challenge in data citation. This leads to the need for an additional level of identifier, something that would be able to aggregate all the project level information of component images while also maintaining the item level identifiers. This was designated a functional level. It could be created from a search results page, in order to ensure that any required review or modification of a result set could be done before the final identifier is assigned in order to avoid repeated unnecessary creation of identifiers. Once created, it would provide a way to directly access the specific image set being cited, as well as identify the origins of the individual images and their creators. Figure 1 shows a schematic overview of this approach and workflow.

One consideration when allowing for the creation of functional level identifier is avoiding duplication. In the prototype implementation of this system, described below, any time a result set is tagged with an identifier, it is compared to a local record of all other created functional level identifiers and their component images—if identical results sets are tagged, the returned identifier is the same. This reuse of an identifier, however, can then be appended to an extant record, creating space for new and different descriptive information to be added. In this manner, an identical set of images may be labeled as being used in multiple studies, or referenced by multiple publications, but the identifier used is the same. This method allows the breadth of impact of a particular image or dataset to be clearly displayed, with each reuse presented with its own list of contributors, descriptive information, and related citations.

### How to cite?

After investigating the possible citation and identification schemes currently in use in various scientific communities, such as RRID (Bandrowski et al., 2016)^14^, Thomson Reuters PermID^15^, PURL^16^, Handles (Ball and Duke, 2015)^17^, we determined that the DOI^18^ is the most broadly accepted and most widely supported. A DOI can be associated with each image, and subsequently grouped into collections which themselves can be assigned a DOI (and associated with authors, publications, or grants). This scheme fulfills the need for establishing credit by maintaining image level attribution within collections. By using the EZID^19^ system to assign DOIs, the DataCite Metadata Schema^20^, V. 3.1 metadata standard can be applied articulating the minimum amount of documentation required when uniquely identifying any individual data element (in this case, an image or dataset). The five required fields that must be present for every instance of identifier creation are: identifier, creator, title, publisher, and publication year. Other fields are suggested, and provide more complete contextualization, but may be omitted. Table 1 reviews the proposed usage of the DataCite Metadata Schema in the context of neuroimaging data sharing. While similar in construction, there are some important differences in the schema description for image, project or functional identifiers.

**Table 1:** **Fields and subfields defined by DataCite Metadata Schema V 3.1 as are field level requirements**.

	**Field/*Subfield***	***M/R/O***	**Description**	**Image level**	**Project level**	**Functional level**
**1**	**Identifier**	M	The identifier is a unique string that identifies a resource			
**2**	**Creator**	M		Original creators	Original creators	Creators of data included in collection
2.1	*creatorName*		The main researchers involved in producing the data, or the authors of the publication, in priority order.			
2.2	*creatorIdentifier*		ORCID identifier			
2.3	*creatorIdentifierURI*		URI of identifier scheme used			
2.4	*Affiliation*		affiliations are from the time of the data creation/paper publication			
**3**	**Title**	M	A name or title by which a resource is known			
**4**	**Publisher**	M	The name of the entity that holds, archives, publishes, prints, distributes, releases, issues, or produces the resource. This property will be used to formulate the citation, so consider the prominence of the role			
**5**	**PublicationYear**	M	The year when the data was or will be made publicly available			Year functional DOI assigned
**6**	**Subject**	R	Subject, keyword, classification code, or key phrase describing the resource. Free text			Can also include search terms/query used to produce collection
6.1	*subjectScheme*		i.e.,: MeSH terms from publication			
6.2	*schemeURI*		URI for souce of scheme			
**7**	**Contributor**	R	The institution or person responsible for collecting, managing, distributing, or otherwise contributing to the development of the resource			Creator of the collection
7.1	*contributorType*		contact person, funder, etc.,			
7.2	*contributorName*					
7.3	*Affiliation*					
**8**	**Date**	R	Different dates relevant to the work.			
8.1	*dateType*		Accepted, Available, Copyrighted, Collected, Created, Issued, Submitted, Updated, Valid, etc.,			
**9**	**Language**	O				
**10**	**ResourceType**	R	A description of the resource			
10.1	*resourceTypeGeneral*		ex: Dataset/Imaging Data	Image/Structural MRI Image	Dataset/Imaging Data	Dataset/Imaging Data
**11**	**AlternateIdentifier**	O	An identifier or identifiers other than the primary Identifier applied to the resource being registered			
11.1	*alternatIdentifierType*		The type of the AlternateIdentifier			
**12**	**RelatedIdentifier**	R	Identifiers of related resources. These must be globally unique identifiers			Can be iterated if the same data collection is used by multiple publications, papers should include a DOI/References as part of the data description
12.1	*relatedIdentifierType*		DOI, PMID, etc.,			
12.2	*relationType*		*HasPart, IsSourceOf, IsReferencedBy, IsDocumentedBy*			
**13**	**Size**	O	Unstructured size information about the resource			
**14**	**Format**	O	Technical format of the resource			
**15**	**Version**	O	The version number of the resource			
**16**	**Rights**	O	Any rights information for the resource			Inherits from source rights, list of all
16.1	*rightsURI*		The URI of the license			
**17**	**Description**	R	All additional information that does not fit in any of the other categories. May be used for technical information			
17.1	*descriptionType*		Abstract, Methods, SeriesInformation, TableOfContents, Other			
**18**	**GeoLocation**	R	Spatial region or named place where the data was gathered or about which the data is focused		Need to consider PHI implications	
18.1	*geoLocationPoint*					
18.2	*geoLocationBox*					
18.3	*geoLocationPlace*					

M, Mandatory; R, Recommended, O, Optional.

The context of this approach to data identification and credit defined three levels of data and their appropriate identifiers in the following way: The most basic unit of data to be identified is a single scan or image, which we call an **image level** identifier. All of the images produced and published by a single study, or used as the basis for a publication are grouped together as a dataset or collection, and given a project level identifier. Project level DOIs are assigned at the time of upload of the collection into the database or repository that will be responsible for assigning identifiers. The third kind of identifier, a functional level identifier, is assigned to any new collection or grouping of images produced through a database. These can include groupings that are subsets of images from a single project, or include images from multiple originating projects.

### Reference implementation

**Figure 2:**
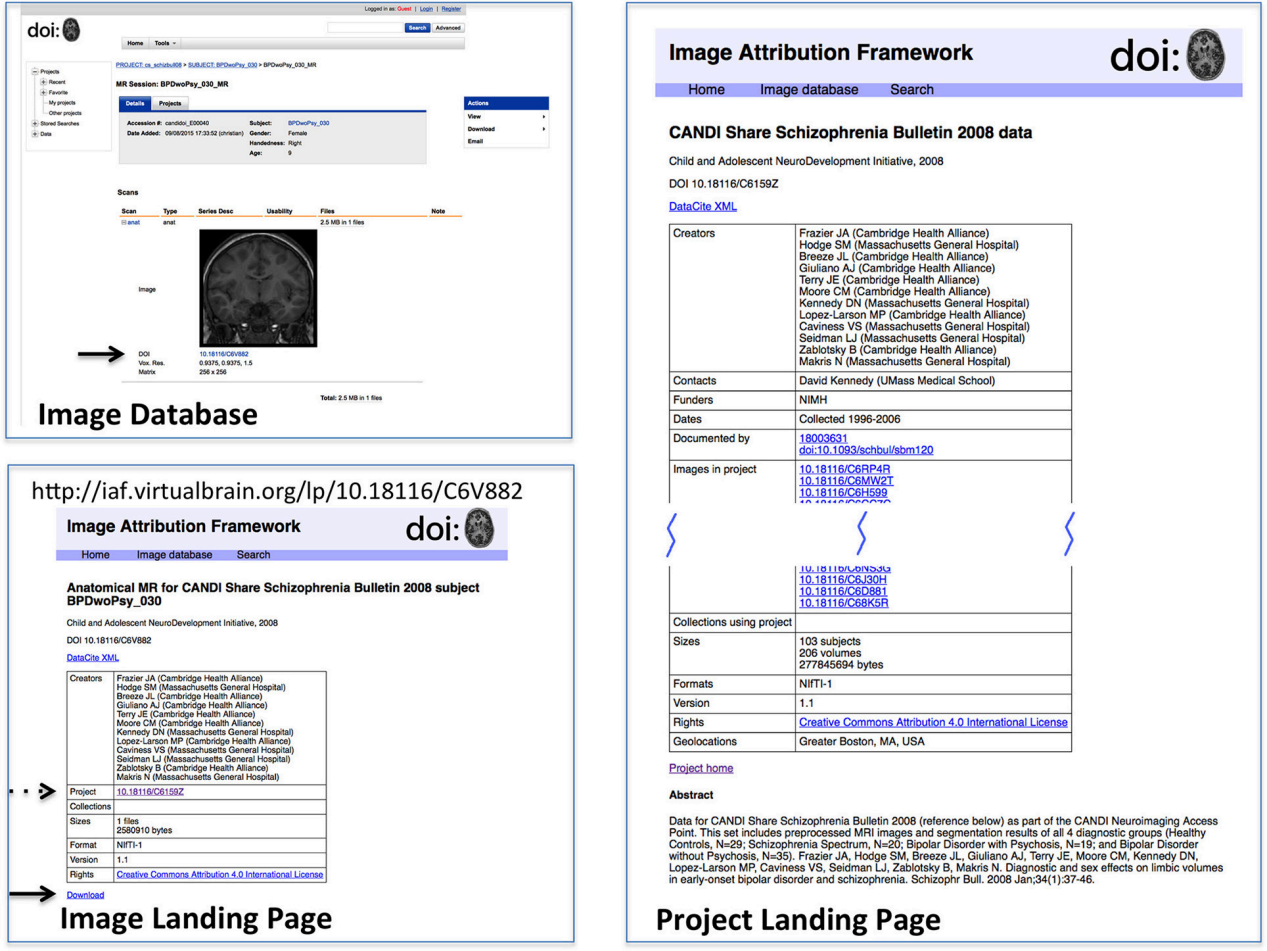
**Reference implementation of DOI-enabled Image Database**. The image database in this implementation is an XNAT instance customized to store DOIs for projects and image data. DOIs link (via dx.doi.org) to landing pages for the objects, which can then link back to the image database. DOI's are generated upon data upload into the database. This example shows the image (upper left) in its database representation (including DOI, arrow); the Image Landing Page (lower left) which is referenced by the image DOI, from which the one can access the download for this image (solid arrow) and find the project citation for this image (dashed arrow); and the Project Landing page (right) associated with this image. Landing pages for projects and images have similar considerations as landing pages for image collections. While projects and images do not need as intricate bookkeeping as image collections, additional semantics do still need to be applied. Images, for instance, use relatedIdentifier with relationType “IsPartOf” for both projects and image collections, so the landing page must keep track of which are which.

**Figure 3:**
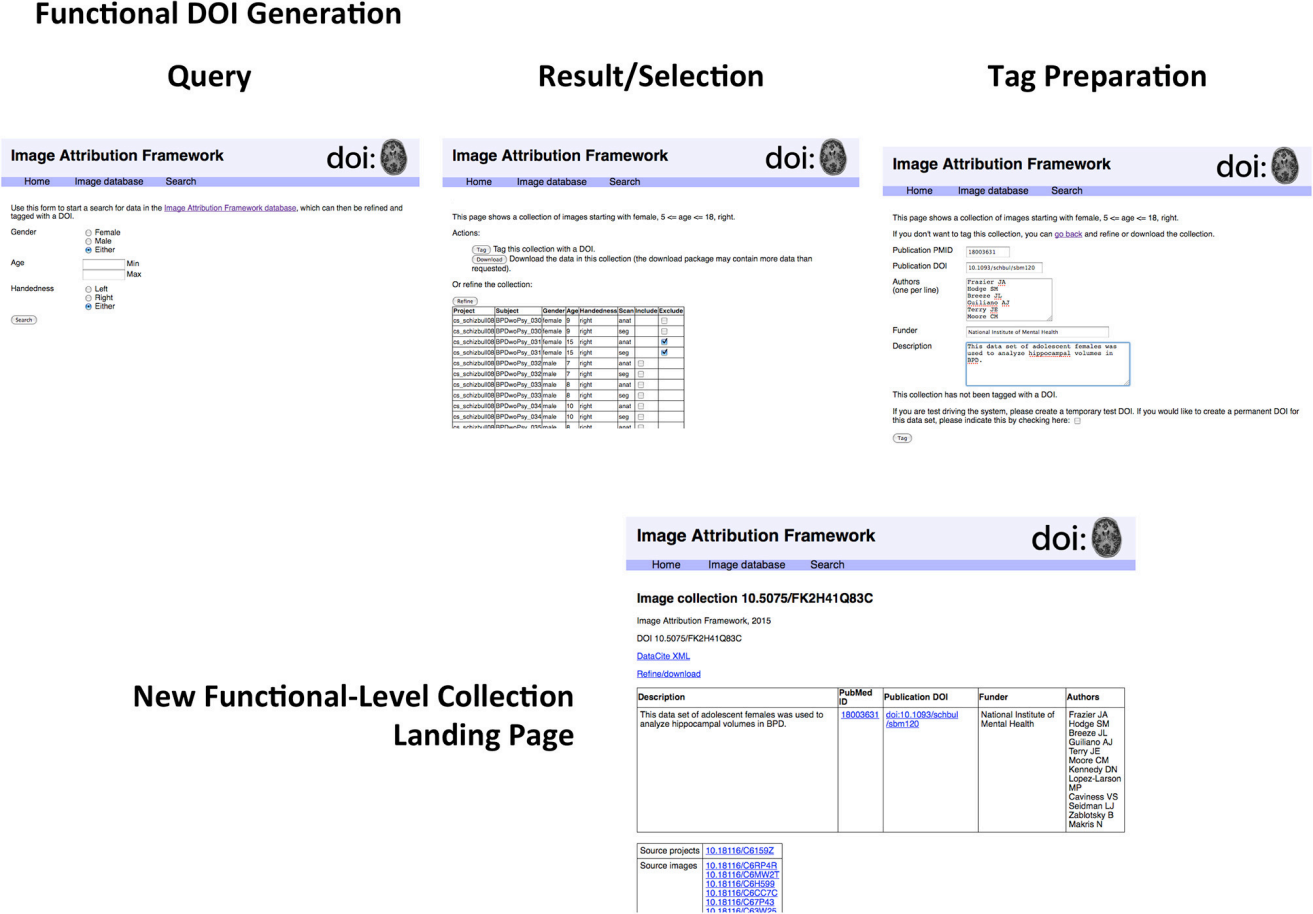
**Generation of functional-level collections**. The sequence of steps includes the following. Search form: The search form mirrors the metadata descriptors of the image database. Examination of search results: The results page allows for finer control in specifying search results for final collection generation. Image collection tag form; Once a precise dataset has been selected, it can be tagged with a DOI. The proof-of-concept implementation allows for creating test DOIs for demonstration purposes. Functional-level Image collection landing page; The resulting image collection with user-specified metadata about the collection. If this collection is arrived at by another route (i.e., if somebody investigating something else happens to work with the same collection of data), a new DOI is not assigned, but the existing DOI is amended. As described in the text, the structure of the landing page does not reflect that of the underlying DataCite representation. A second use of this functional collection would result in another row in the top table; the DataCite schema does not accommodate the grouping of elements in this way.

In order to test this scheme, a sandbox database (Image Attribution Framework) was created, mirroring the structure of the Neuroimaging Informatics Tools and Resources Clearinghouse (NITRC) neuroimaging data repository using the XNAT^21^ (Marcus et al., 2007) platform. The EZID API was integrated in order to allow DOIs to be assigned upon dataset upload, meaning that both an overarching (project level) identifier is attached to the set as a whole, and each image within that set had its own DOI (image level) which reflects its individual attributes, as well as inheriting provenance information from its parent project. Suggested data citations could be automatically produced for either an item or a project, and the DOIs allow for machine readable identification of the cited data. This proof-of-concept implementation is publicly available at iaf.virtualbrain.org. Figure 2 shows an example of image and project level identifiers. Figure 3 demonstrates the generation of a functional level identifier from the results of a database search including each of the component elements of this implementation. Note that our reference implementation is for illustration purposes. It is expected that ultimate success of this concept will be through the implementation of these recommendations within the many existing (and any new future) data hosts, and not through this reference implementation itself.

### Landing pages

While DOIs allow for the unique identification of images and datasets, it is the corresponding landing pages that allow for understanding of what exactly the DOI is identifying. The landing page is a human-readable web-page that resolves from the machine-readable DOI, and must be comprehensive enough to provide the contextualizing information about the image or dataset required to make it useful or reusable. Figures 2, 3 show example image landing pages for this proposed implementation.

## Discussion

In this paper, a set of implementation recommendations for data citation in the context of neuroimaging data that fulfills the joint requirements of documentation, citation, and credit is provided.

### Practical issues

Given the above prescription for data citation, the impact of the implementation of this system was examined for both the investigator and the various data hosting facilities.

#### Researcher perspective

For each publication produced, a researcher will need to both select a method for sharing the data, and have DOIs generated for both the overall data collection used in the publication (project-level) and the individual scans (image-level) of that collection. How these identifiers would be generated is dependent upon the selection of data sharing platform. In general, there are three broad types of hosting options: central repositories (such as NITRC-IR (Kennedy et al., 2016)^22^, COINS Landis et al., 2015,^23^, LONI IDA Crawford et al., 2015,^24^, XNAT Central Marcus et al., 2007,^25^, etc.), institutional repositories (such as eScholarship@UMMS), or a personal repository (on an individual or laboratory website). In order to assign project and image DOIs, a partnership with EZID is required. DOI generation requires the information described in Table 1 to be specified, so the researcher must organize this information according to the DataCite schema. Single DOIs can be created by entering this data into a web form; web services also exist to create DOIs when this data is prepared in a pre-defined machine-readable form (EZID accepts XML and JSON for this purpose). Our reference implementation uses this second database-mediated API approach for our proof-of-concept system. All of the necessary metadata is managed by the local database (XNAT in this case) so that project and image DOIs are generated and cross-linked automatically.

#### Data hosting perspective

The impact of this proposal on a data host is twofold: (1) related to operational functions of a data host (assign the DOI, collect the metadata, provide a landing page, etc.); and (2) related to data host policy (backup procedures, sustainability plans, resource sunsetting contingency, etc.). It is anticipated that it will become the responsibility of the hosting site (central, institutional, or personal) to manage the generation and dissemination of the DOIs. Hosting sites will be responsible for ingestion of the necessary metadata and execution of the DOI generation process. The use of structured data management systems (such as XNAT) in the context of higher-level project information (such as provided in NITRC-IR) can simplify the metadata management, compared to potentially more *ad-hoc* solutions that would have to be managed for personal hosting. The requirement for DOIs to be permanent places additional demands on data hosting facilities. Data hosts, similar to publications, should have adequate backup procedures in place, as well as contingency plans should the host cease operations in the future.

#### Cost

The cost per DOI is miniscule. Annual DOI creation costs range from $500 (for non-degree granting departments) to $25,000 (for entire degree-granting institutions) per 1 million DOIs minted. However, we do appreciate that this proposed scheme will result in the generation of many DOIs per study (in order to index the individual scan level), and propagate DOIs and their provenance, as derived works are generated and then shared themselves. At one extreme, all images from all imaging sessions of all subjects in a study funded by a mechanism that requires all data to be (eventually) shared could receive a DOI, as in principle each of those images may be accessed and used in a subsequent study, for which the original investigator, funder, and data host would all like their credit. On the other hand, data elements that have the potential to be shared or used in a publication may not need a DOI until they actually are shared or used, in which case, one can imagine a more “on demand” generation of DOIs for various data elements. Each of these DOI-related issues places demands on the data host, which will be required to generate and index these identifiers.

#### Demand and uptake

There are a large number of publications that are now catering to the publication of data. The journal *Neuroinformatics* has an “Original Data Article” publication type; the journal *Scientific Data* features the publication of a “Data Descriptor” publication and has a special section on Human Brain MRI Reproducibility^26^; Elsevier's “Data in Brief” and many other examples of data-specific publication exist. The proliferation of these data publications accelerates the need for unique data element specification as this should lead to more demand for data sub-setting and attribution thereof. Each of the existing data publications relies on specification of dataset landing pages. Adoption of a unified scheme across the numerous data hosting options within a community will be important to streamline the data publication process and lessen the burden on data authors.

#### Landing pages

While it might seem more efficient to have a DOI resolve to the data itself (in whatever repository or database its creators choose for deposit) this can lead to issues with preservation, access permission management, and long term reuse. A landing page with well-developed metadata can provide information on what the data was, even if it has been moved, updated, or is no longer available. These “tombstones” are always going to be preferable to broken links or dead ends. Even if the data can no longer be accessed, at least information about what it was can still be found.

Additionally, landing pages allow more flexibility than merely directing someone to the desired data. These can be customized in order to reflect the many types of data and their various conditions: they can provide direct access to data that is open and unregulated or instructions for requesting necessary permission to access data that is not. They can show related citations in the form of publication DOIs or PubMed ID numbers and link to all of the other datasets with shared components. Landing pages can be updated with new relational information or redirect when data is moved between hosts. The DataCite Metadata Schema provides guidelines outlining the minimum necessary information for identification, but much more can and should be included on the landing pages in order to make understanding and reusing the data easy and efficient.

Indeed, it was found that the landing pages must provide additional value beyond what is captured in the DOI metadata. The DataCite metadata fields are broadly defined and in many cases publication-centric. Adapting these fields to neuroimaging data means interpreting fields in a certain way and often overloading fields. For example, the RelatedIdentifer field with type IsPartOf must be used to refer to both the source (project-level) and each collection (functional-level) that the image belongs to. The landing page must make the distinction between the types of IsPartOf RelatedIdentifier, so it (or its underlying system) must provide additional bookkeeping above and beyond what is provided by the DataCite schema. This will be true of any specific subject area to which the general DataCite schema is applied. We have noticed, within the domain of neuroimaging, that the landing pages currently being implemented in the afore-mentioned data publications are quite variable and include minimal support for attribution of individual datasets within any given data release. Currently, there is a distinction between the publication DOI and the data identifier that is associated with that publication. However, current data identifiers can take several different forms (DOIs, database accession numbers, data file URL, etc.), and in most of these examples for neuroimaging data, the data DOI references many individual images, often from different sources, that cannot be de-referenced to the component images. By including individual constituent image DOI's as part of the data DOI, we can achieve a finer-grained attribution, particularly when subsets of this original data release are reused. Table 2 shows the variability one encounters in just a few current existing examples of MRI dataset landing pages, in the context of the key data descriptors required to form a proper bibliographic citation. Future efforts to standardize the content of landing pages, and ability to identify constituent data should continue to be pursued with the various data hosting enterprises.

**Table 2:** **Landing page comparison**.

**Publication**	**Publication DOI**	**Data identifier**	**Download Link**	**Data Creator/Author**	**Data Title**	**Data Publisher**	**Data Publication Year**
Zuo et al., 2014	10.1038/sdata.2014.49	Includes 31 separate data DOI's					
		10.15387/fcp_indi.corr.jhnu1 (Supplementary Figure S1)	✓	?^a^			
		10.15387/fcp_indi.corr.ipcas4 (Supplementary Figure S2)	✓	?^a^^,^^b^	?		
		10.15387/fcp_indi.corr.uwm1 (Supplementary Figure S3)	✓	?^a^			
Hanke et al., 2014	10.1038/sdata.2014.3	https://openfmri.org/dataset/ds000113/ (Supplementary Figure S4)	✓	?^a^			
Watson et al., 2016	10.1016/j.dib.2016.03.100	Data is provided as a supplementary table and a download zip file, with no clear landing page (Supplementary Figure S5)	✓	?^c^			

a Are “Principal Investigators” synonymous with authors?

b Are individuals listed in the “Acknowledgements” synonymous with authors?

c Are the data authors the same as the data article authors?

In the same way, we keep track of and group the metadata associated with each approach leading to (corresponding to each meaning of) a functional-level data collection. Identical data sets have only one DOI and one set of metadata. The DataCite schema does not allow association with a given description with a PubMed ID; all of the descriptions are stored together (in a simple list of Description fields) and all PubMed IDs are stored as RelatedIdentifiers with relatedIdentifierType PMID (also alongside all of the RelatedIdentifiers of other types, such as DOI) in another simple list. Similar embedding is also needed for publication DOIs, funders, and authors. It is therefore incumbent on landing pages to keep track of and display which descriptions go with which PubMed IDs, publication DOIs, funders, and authors. This is key to the system and expressing the different meanings of functional-level datasets that must have a unique DOI. This is not, however, a weakness of the system, since the researchers have just seen that the DOI metadata is semantically incomplete when applied to any specific subject area and must be understood and interpreted separately from the DOI system.

### Assessment

At the start of this initiative, it was identified that the researchers wanted an identification and credit system that would (1) uniquely identify data; (2) provide a method for citation; and (3) ensure that the chain of attribution is maintained as the data is used and reused. The proposed system easily satisfies objective 1 through the guaranteed uniqueness and permanence of the DOI. The ultimate location of the data records themselves can change, but the landing page for the DOI can reflect such a change. Even data that becomes “lost” (through catastrophe or lack of use or other event) will have a record of its existence and subsequent circumstances of its demise.

Objective 2, attribution, is also facilitated by the use of the DOI, at both individual (Frazier et al., 2008a) and collective levels (project: Frazier et al., 2008b; functioanl collection: Breeze et al., 2016), through the pre-existing utilities designed to track publication citations. One of the main benefits for using DOIs explicitly is the existing infrastructure for monitoring, tracking and indexing their use. Given publications that are annotated with the input data DOIs, citing them in the text and including them in the references, the existing tracking systems will provide a means to derive credit and usage reports. This includes a “citation count” (h-index for a dataset) for documenting how often a specific unique data set is used in publications and a dataset “provenance” which can be tracked through the literature. Enterprises such as CrossRef^27^ and the Data Citation Index^28^ facilitate such information use management, and are easily accessed once DOIs are generated and registered with these organizations. There is some added complexity that needs to be handled when citation to a collection requires assignment of credit to each of the individual images in that collection. In this case, the data host for each image is required to provide support for the tracking of all the collection DOIs that their individual images are part of. Then the data host can aggregate the citations for this set of DOIs in order to generate a total citation count for an image^29^.

Individual data sets are expected to be reused in derivative publications, but the source data for these future publications can be expected to potentially mix and match data from numerous and disparate sources. Objective 3 aims to ensure that credit for the individual images that comprise hybrid data sets is correctly and accurately attributed while minimizing the overhead involved in providing this tracking information by the subsequent data user. The complete dataset of a subsequent publication will receive its own DOI, but will also reference all the DOIs of the constituent data, permitting both high and low level use tracking and credit attribution.

In summary, we feel the proposed system successfully met the design objectives.

### Relationship to other approaches

We believe that our “functional DOI” scheme meets the general principles of RDA^30^ Working Group on Data Citation's^31^ recommendations. We have adapted the implementation in a number of ways as needed to support the current status of the neuroimaging data hosting community. Since the data hosts do not expose all possible features of the data to their queries, our proposed system allows for post-search refinement of search results. The search semantics central to the Working Group's recommendations are captured in the functional-level DOI. This allows for describing post-search adjustments to the search results (i.e., exclusion of data based on quality metrics or associated subject demographic features), and also allows for capture of the semantics describing the search. The driving motivation to this scheme is to identify a data set as it was used in an analysis, which will facilitate replication of studies. We expand upon the RDA recommendation by requiring all constituent images to have DOIs, and for these DOIs to be enumerated in the resultant collection. This extension is required since, for the time being at least, there are numerous different data sources that provide data that can be combined to generate new data collections.

### Future directions

The assigning of DOIs and their use in citations of both original datasets and derivative sets used in new research allows for the tracking and quantitative measuring of not only publication impact, but data-specific impact. As individual images are reused and included in new functional level DOIs, their project level landing pages reflect those instances of citation. Once data identifiers become a standard requirement, inclusion in commercial indices, such as the Thompson Reuters Data Citation Index allow for increased discoverability. Data citations could be included in the calculation of impact factors for journals, publication altmetric measurements, as well as author-specific metrics.

This case study illustrates that it is possible to automate the creation of DOIs to facilitate the appropriate reuse of data and acknowledgement of credit within the neuroimaging community. While it was effective within the small, locally managed database, further testing in larger, more complex environments will be needed.

One consideration when applying this system to neuroimaging data as a whole is the duplication of identifiers. While the researchers had the luxury in this prototype of a small collection of data under local control, there are several existing repositories, and many data sets. Care must be taken to avoid duplication of DOIs where a single data set exists in or is exposed by multiple repositories. The solution to this problem is not obvious, but it must be addressed. Effort on the part of the author will be necessary to monitor where their primary data is shared, and to make sure that co-hosting of the primary data also is referenced to a common identifier. Similarly, functional collections must be globally unique, so repositories cannot simply act on their own to create functional-level DOIs as our prototype system does. This problem is compounded when functional collections contain images from multiple repositories. As data hosting options proliferate, it is expected that the community will next see numerous “aggregator” sites (sites that aggregate data from multiple sources, such as NIF Gardner et al., 2008,^32^or the Child Psychiatry Portal Rane et al., 2014) which will need to monitor and propagate the appropriate identifiers.

Another issue for future consideration is standards for landing pages. The DOI specification does not impose requirements or standards for landing pages, and this is appropriate to its scope. In practice, landing pages vary in their quality and usefulness, and it will fast become an issue that needs to be addressed. At the very least, required or recommended data to be included on landing pages could be addressed. Semantic structures for subject-specific entities (such as our functional collection tag, which groups a description, a publication, authors, and a funder) can be specified as necessary for various disciplines. This need not rise to the level of a standard format or look and feel to a landing page—indeed, institutional branding, and some freedom in the structure of the page is important—but having a common expectation of what will be on a landing page and a pre-existing understanding of the subject-specific data that is found there will be valuable.

## Conclusion

Here, we have presented a system for neuroimaging data citation and credit with a practical implementation that meets the objectives of unique identification, data use tracking, and integration with traditional credit attribution systems. While the implications for the way researchers engage in their publication and post-grant activities are not trivial, changes in these data sharing and crediting practices are necessary for the neuroscience field as a whole, in order to advance the goals of reproducible science.

## Information sharing statement

The proof-of-concept implementation is accessible at http://iaf.virtualbrain.org. The software code for the implementation of the proof-of-concept is available at https://github.com/chaselgrove/doi.

## Author contributions

All authors LH, CH, JF, and DK contributed to the design, writing, and approval of this manuscript, and are accountable for its content.

### Conflict of interest statement

The authors declare that the research was conducted in the absence of any commercial or financial relationships that could be construed as a potential conflict of interest.

## Footnotes

^1^http://www.nature.com/sdata/collections/mri-reproducibility

^2^https://rd-alliance.org/system/files/documents/RDA-DC-Recommendations_151020.pdf

^3^http://www.force11.org/group/joint-declaration-data-citation-principles-final

^4^http://adni.loni.usc.edu/

^5^https://ndar.nih.gov/

^6^http://fcon_1000.projects.nitrc.org/

^7^http://fcon_1000.projects.nitrc.org/indi/abide/

^8^http://fcon_1000.projects.nitrc.org/indi/adhd200/

^9^“*Open data is data that can be freely used, re-used and redistributed by anyone—subject only, at most, to the requirement to attribute and sharealike.”*

^10^http://www.nitrc.org/

^11^http://datadryad.org/

^12^https://figshare.com/

^13^http://escholarship.umassmed.edu/

^14^https://scicrunch.org/resources

^15^https://permid.org/

^16^https://purl.oclc.org/docs/index.html

^17^http://handle.net/index.html

^18^https://www.doi.org/

^19^http://ezid.cdlib.org/

^20^https://schema.datacite.org/

^21^http://www.xnat.org/

^22^http://www.nitrc.org/ir/

^23^https://coins.mrn.org/

^24^https://ida.loni.usc.edu/

^25^https://central.xnat.org/

^26^http://www.nature.com/sdata/collections/mri-reproducibility

^27^http://www.crossref.org/

^28^http://thomsonreuters.com/en/products-services/scholarly-scientific-research/scholarly-search-and-discovery/data-citation-index.html

^29^Our proof of concept Image Attribution Framework does not include this functionality. It can be argued that this functionality does not belong with the image databases but rather with separate services that operate on the DOIs that are generated by these databases.

^30^https://rd-alliance.org/

^31^https://rd-alliance.org/groups/data-citation-wg.html

^32^http://neuinfo.org/
