# Corrigendum: Data Citation in Neuroimaging: Proposed Best Practices for Data Identification and Attribution

**DOI:** 10.3389/fninf.2016.00043

**Published:** 2016-10-05

**Authors:** Leah B. Honor, Christian Haselgrove, Jean A. Frazier, David N. Kennedy

**Affiliations:** ^1^Lamar Soutter Library, University of Massachusetts Medical SchoolWorcester, MA, USA; ^2^Eunice Kennedy Shriver Center, University of Massachusetts Medical SchoolWorcester, MA, USA; ^3^Child and Adolescent NeuroDevelopment Initiative, Department of Psychiatry, University of Massachusetts Medical SchoolWorcester, MA, USA

**Keywords:** data citation, data attribution, credit, data repository, data sharing

In our original article, we stated, “Annual DOI creation costs range from $500 (for non-degree granting departments) to $25,000 (for entire degree-granting institutions) per 1 million DOIs minted.” This is incorrect. The University of California EZID service offers annual DOI creation at $835 (for non-degree granting departments) to $2500 (for entire degree-granting institutions) per 1 million DOIs minted^1^. The authors apologize for the mistake. This error does not change the conclusions of the article in any way.

## Author contributions

All authors contributed to the design, writing, and approval of this manuscript, and are accountable for its content.

## Funding

This work was supported by NIH grants R01 MH083320 (NIMH and NLM) and P41 EB019936 (NIBIB).

### Conflict of interest statement

The authors declare that the research was conducted in the absence of any commercial or financial relationships that could be construed as a potential conflict of interest.

## Footnotes

^1^http://ezid.cdlib.org/learn/

