# Supplementary material for: Data Citation in Neuroimaging: Proposed Best Practices for Data Identification and Attribution
Source: Front Neuroinform. 2016 Aug 12;10:34. doi: 10.3389/fninf.2016.00034 (PMC4981598; doi:10.3389/fninf.2016.00034)

**List of Supplementary Figures**

**Supplementary Figure S1.** Landing page for 10.15387/fcp_indi.corr.jhnu1

**Supplementary Figure S2.** Landing page for 10.15387/fcp_indi.corr.ipcas4

**Supplementary Figure S3.** Landing page for 10.15387/fcp_indi.corr.uwm1

**Supplementary Figure S4.** Landing page for https://openfmri.org/dataset/ds000113/

**Supplementary Figure S5**. Landing page for data associated with 10.1016/j.dib.2016.03.100

Supplementary Figure S1. – Landing page for 10.15387/fcp_indi.corr.jhnu1


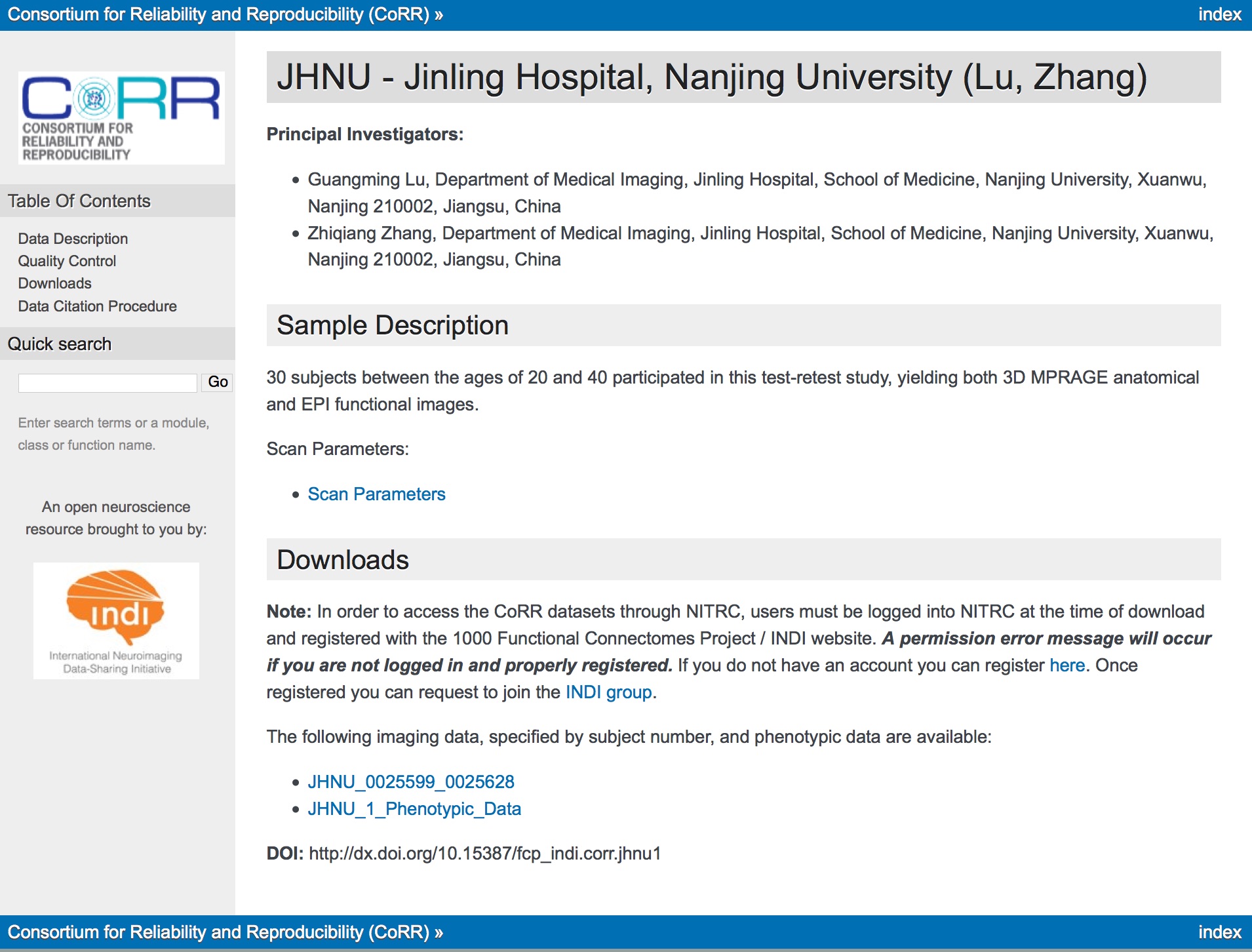


Supplementary Figure S2. – Landing page for 10.15387/fcp_indi.corr.ipcas4


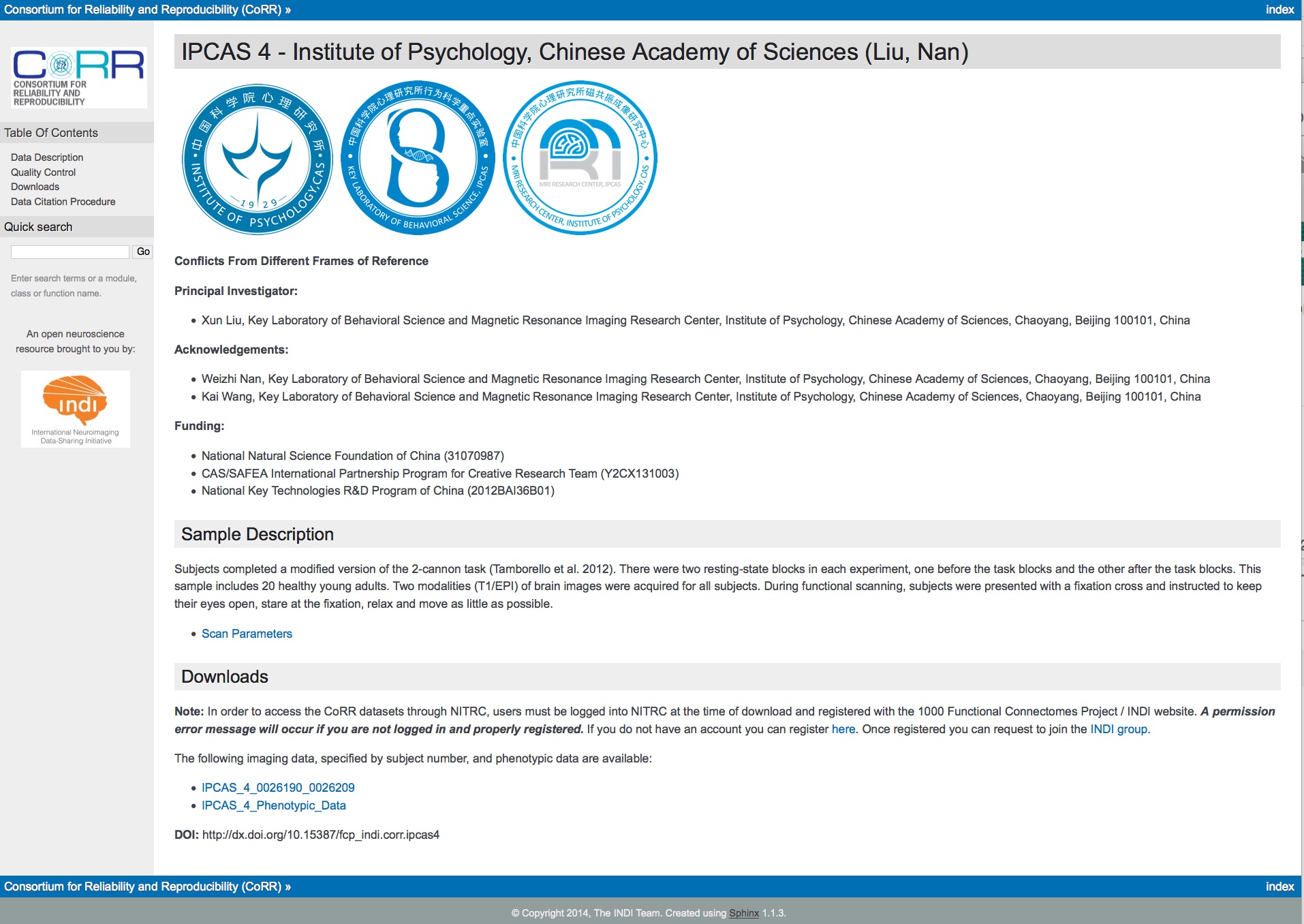


Supplementary Figure S3. – Landing page for 10.15387/fcp_indi.corr.uwm1


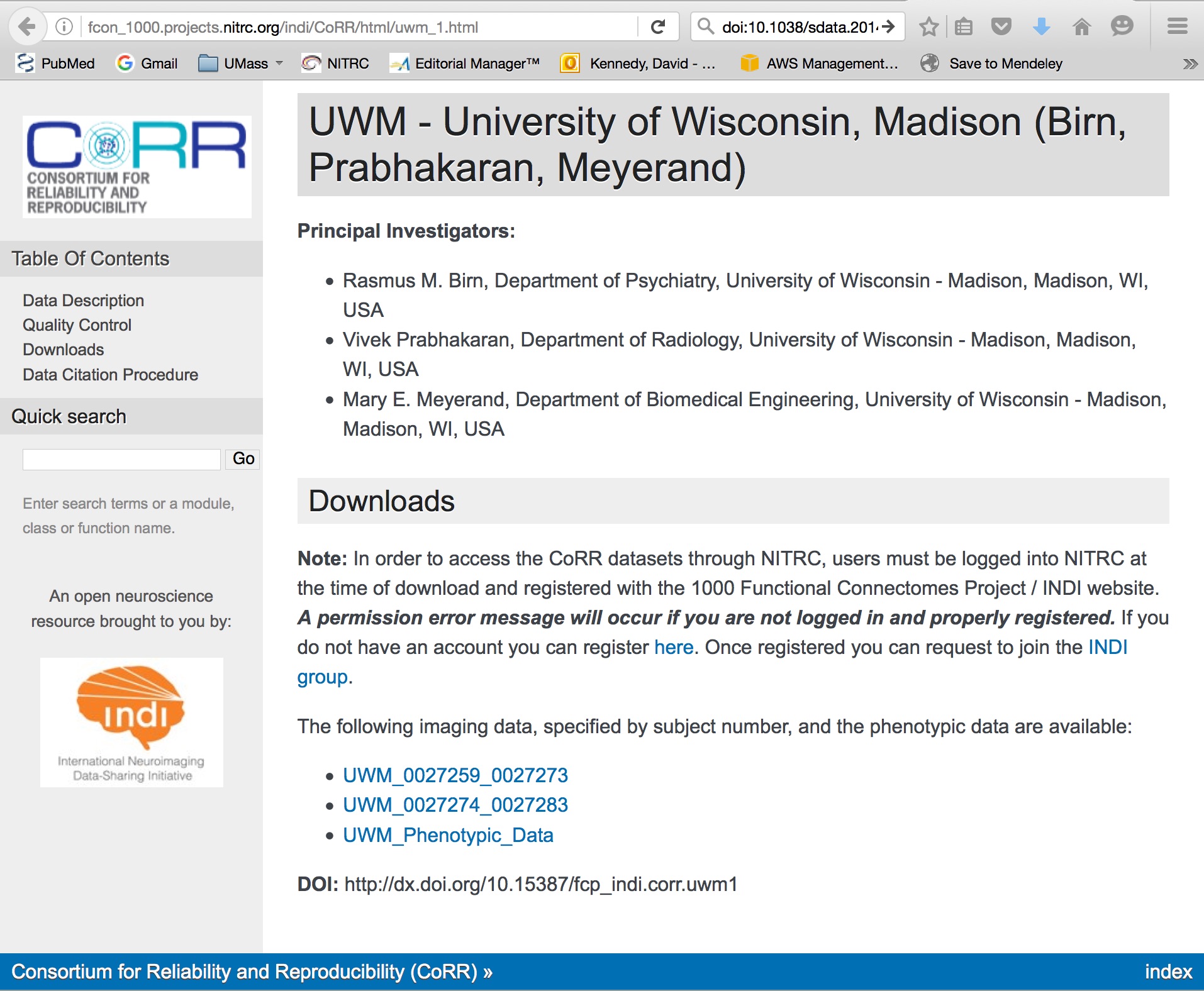


Supplementary Figure S4. – Landing page for https://openfmri.org/dataset/ds000113/


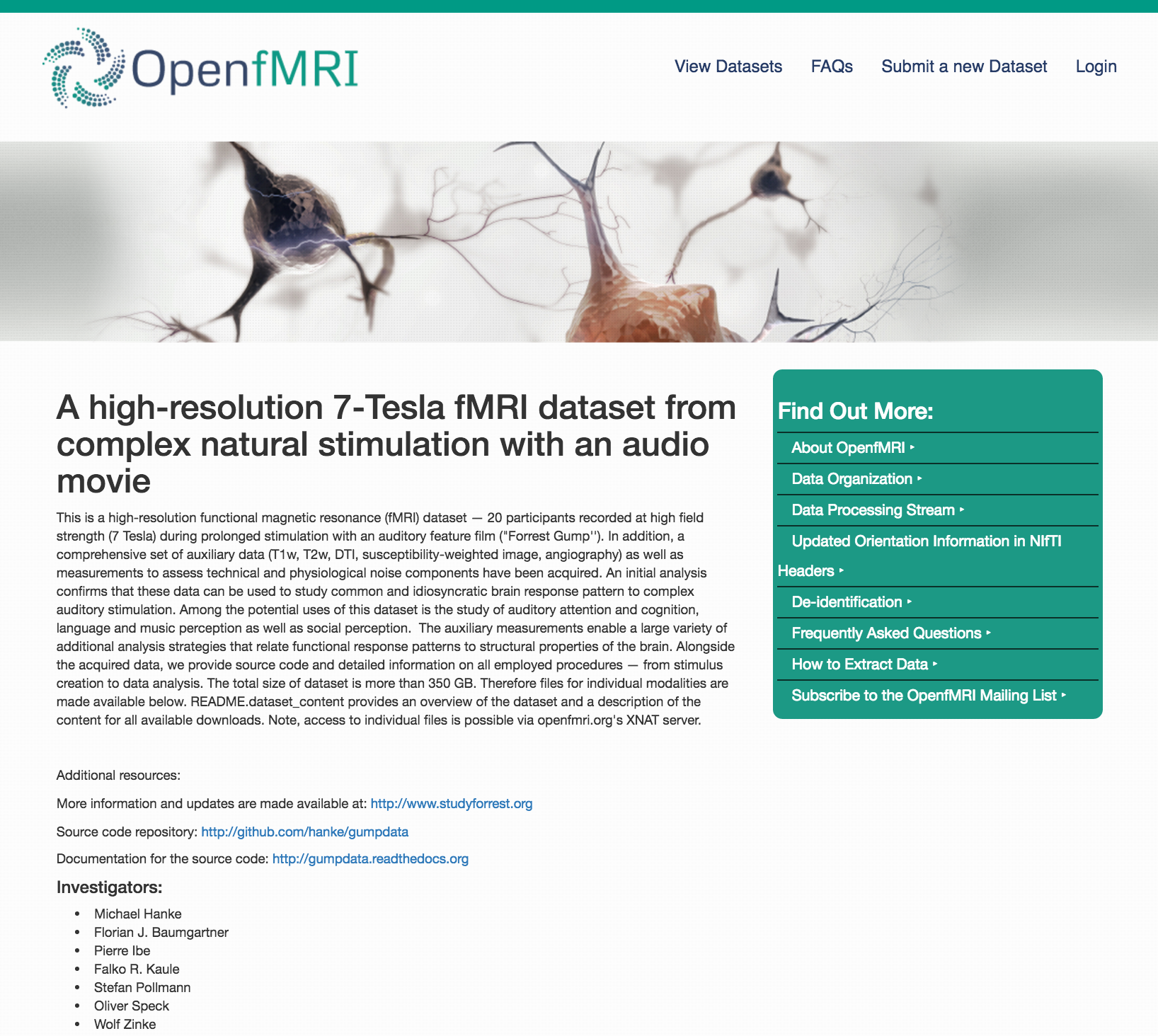


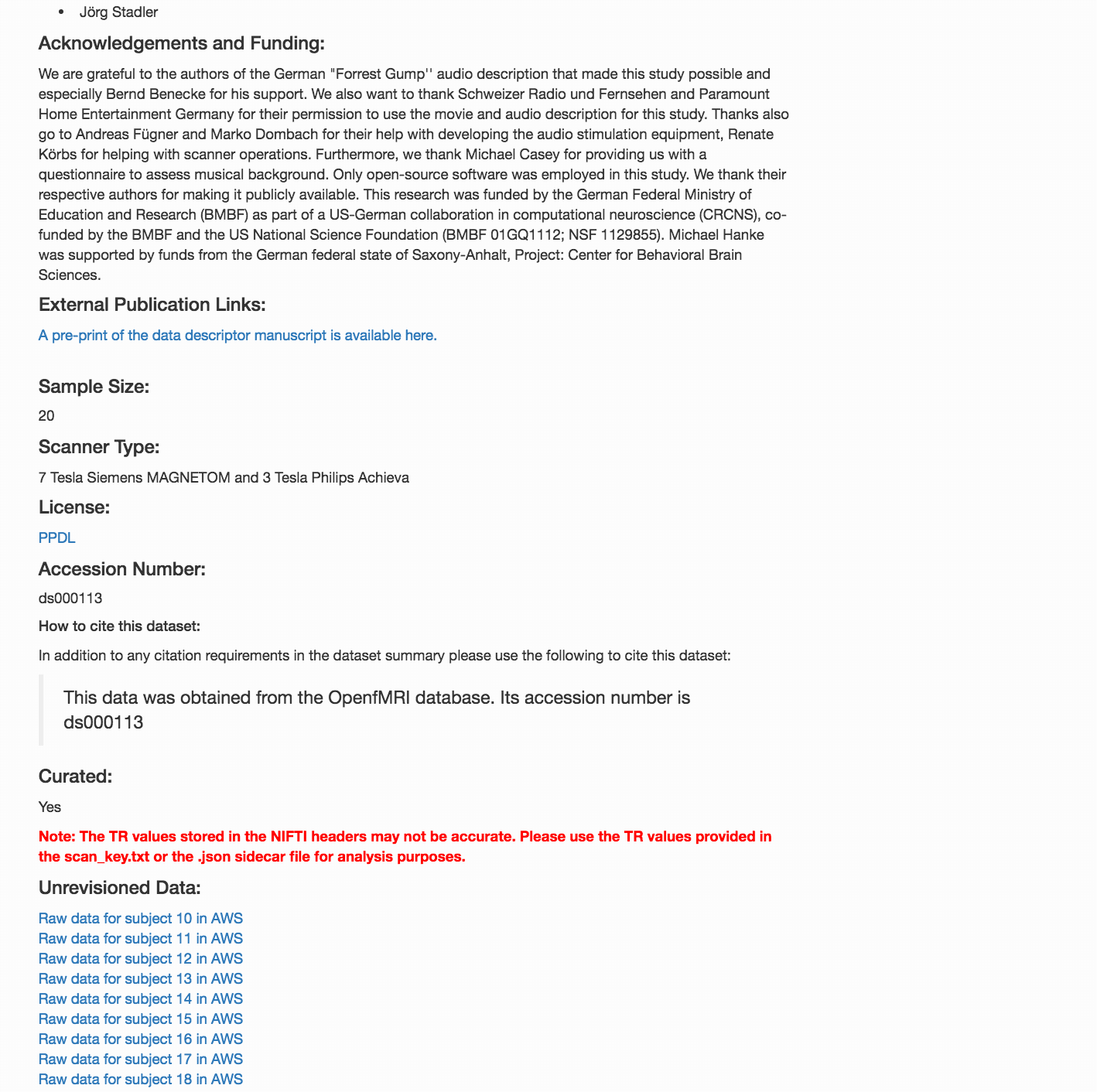


Supplementary Figure S5. – Landing page for data associated with 10.1016/j.dib.2016.03.100


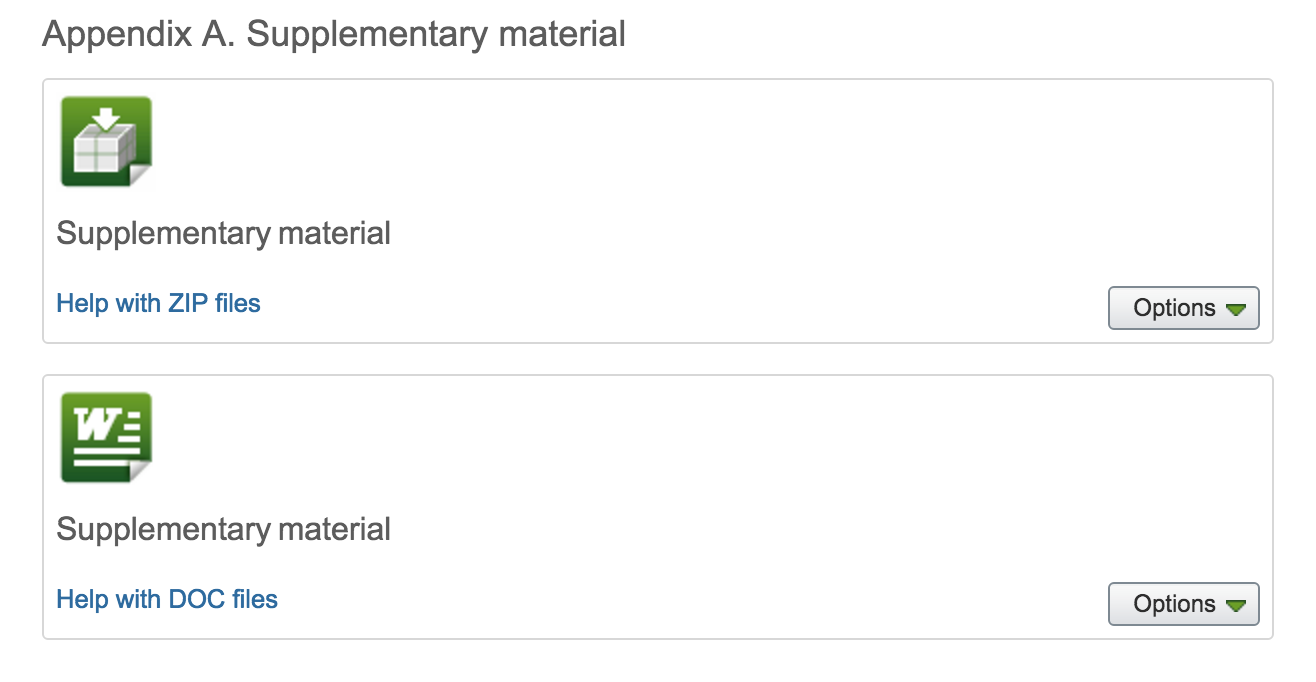

Supplement: Supplementary file 1 [file DataSheet1.DOCX]
